# Machine learning identifies immune-perinatal predictors of infantile hemangioma

**DOI:** 10.3389/fped.2025.1662381

**Published:** 2025-11-03

**Authors:** Dongdong Wu, Neng Wan

**Affiliations:** Department of Burn and Plastic Surgery, The Affiliated Huaian No.1 People's Hospital of Nanjing Medical University, Huaian, Jiangsu, China

**Keywords:** infantile hemangioma, immune-inflammatory marker, machine learning, XGBoost, risk factor

## Abstract

**Background:**

Infantile hemangioma (IH), the most common vascular tumor of infancy, exhibits hallmark features of immune and inflammatory dysregulation. While most cases are self-limiting, a subset progresses with potentially severe complications. Despite its benign classification, IH offers a unique model to investigate immune-mediated mechanisms in early tumorigenesis. However, risk stratification models incorporating immune-inflammatory markers remain underdeveloped.

**Methods:**

A total of 1,466 infants and young children were enrolled, including 81 with IH. Comprehensive perinatal, clinical, and laboratory data were collected. Candidate risk factors were identified using logistic regression. Four machine learning algorithms—XGBoost, Random Forest, Support Vector Machine, and k-Nearest Neighbors—were employed to construct predictive models. Model performance was assessed through internal and external validation. SHapley Additive exPlanations (SHAP) were applied to interpret feature contributions and immune-inflammatory signatures.

**Results:**

Key risk factors included prematurity, multiple gestation, low birth weight, and elevated levels of VEGF, CRP, and SAA—markers linked to inflammation and immune activation. The XGBoost model achieved superior performance, with an AUC of 0.952 (training), 0.935 (internal validation), and 0.870 (external validation). SHAP analysis highlighted SAA, VEGF, and low birth weight as the most influential predictors, reflecting a critical link between innate immune dysregulation and IH development.

**Conclusion:**

This study presents a robust, interpretable machine learning model that leverages immune-perinatal features to predict IH risk. Our findings support the notion that IH may serve as a paradigm for inflammation-associated vascular tumorigenesis, with implications for early detection and personalized intervention strategies in immune-driven neoplasms.

## Introduction

Infantile hemangioma (IH) is the most prevalent benign vascular tumor in infancy, characterized by abnormal localized or diffuse endothelial proliferation, primarily affecting the skin and soft tissues (1, 2). Although most IHs follow a self-limiting course, their subtle onset and delayed clinical manifestation frequently preclude detection at birth. As lesions emerge and enlarge during early infancy, a subset of cases enters a phase of rapid proliferation, potentially resulting in serious complications such as ulceration, bleeding, infection, functional impairment, or even life-threatening events. Additionally, the aesthetic and psychosocial consequences of disfiguring lesions may cause significant emotional distress and hinder social adaptation in early childhood (3–5). Therefore, early and accurate identification of high-risk cases is essential to initiate timely intervention, reduce disease burden, and improve long-term outcomes.

The natural course of IH involves a well-defined proliferative phase—typically between 1 and 6 months of age, with the most pronounced growth occurring within the first 3 months. During this critical window, hemangioma cells exhibit heightened mitotic activity, driving rapid tumor expansion. Failure to recognize and treat high-risk IHs during this early proliferative stage may result in irreversible tissue damage and complications affecting vision, hearing, or organ function (6–9). Thus, early risk stratification plays a pivotal role in preventing progression and preserving healthy development.

Despite its clinical significance, the identification of robust risk factors for IH remains challenging. Current risk assessment tools rely largely on clinical scoring systems or empirical judgment, which often lack sufficient sensitivity, specificity, and scalability across diverse populations (10, 11). These conventional models tend to incorporate only a limited range of variables, failing to capture the complex, multifactorial pathogenesis of IH—an interplay of genetic, immunological, environmental, and intrauterine factors that remains incompletely understood.

Recent advances in machine learning (ML) offer transformative capabilities for disease risk modeling. ML algorithms excel at analyzing high-dimensional data, identifying complex nonlinear interactions, and generating highly predictive models that surpass traditional statistical methods in accuracy and generalizability (12–14). Despite its growing application in clinical medicine, ML has been underutilized in IH research, especially in Asian populations. This gap highlights both the novelty and necessity of applying ML approaches to IH risk prediction in demographically diverse cohorts.

Although numerous studies from Europe and North America have contributed to our understanding of IH epidemiology and clinical predictors (10, 15–17), their applicability to Asian populations is limited by genetic, environmental, and healthcare system differences. As such, developing population-specific predictive models is crucial for advancing precision diagnostics and personalized care in IH management.

The central hypothesis of this study is that an integrated “immune-perinatal signature,” combining perinatal characteristics with serum immune-inflammatory biomarkers, can reliably predict the onset of IH. We curated a large infant cohort comprising both IH cases and controls and applied multiple machine learning algorithms to identify key risk factors and construct a robust, interpretable predictive model. In this study, we prioritized three biomarkers—VEGF, CRP, and SAA—based on strong biological and clinical rationale. First, VEGF-A and its signaling pathway play a central role in angiogenesis, and elevated VEGF levels have been detected in proliferative IH lesions. Several histological and serum studies further suggest that VEGF levels correlate with disease activity, providing direct pathophysiological support for its role as an angiogenesis-related biomarker. Second, CRP is a widely used clinical marker of acute inflammation. IH lesions, particularly when complicated by ulceration or infection, can trigger systemic inflammatory responses and elevated inflammatory markers; more broadly, inflammation is implicated in IH onset and progression, making CRP a useful indicator of host inflammatory status and IH risk. Third, SAA, another major acute-phase protein, has recently been recognized for its roles in immune regulation, inflammatory pathway activation, and tumor microenvironment modulation. Although direct evidence linking SAA to IH is limited, its potential function along the inflammation–immune–angiogenesis axis makes it a compelling candidate biomarker. Additionally, we applied Shapley Additive Explanations (SHAP) to interpret model outputs and identify the principal biological determinants. This study aims to validate our hypothesis, highlighting the potential of machine learning–driven approaches for early risk stratification, informing personalized therapeutic strategies for IH, and advancing mechanistic insights into immune-mediated processes in early tumorigenesis.

## Materials and methods

### Study subjects

This study leveraged clinical data sourced from three tertiary medical institutions in China: Wuxi People's Hospital, Wuxi Second People's Hospital, and Tengzhou Central People's Hospital. Inclusion criteria encompassed: (1) infants aged 0–12 months at enrollment; (2) undergoing vascular lesion screening at birth or during infancy prompted by physical examination findings or clinical symptoms; (3) availability of comprehensive perinatal data, including gestational age, birth weight, and Apgar scores; (4) detailed maternal-infant records, comprising pregnancy-related complications, conception mode, and history of drug exposure; (5) documented family consent for longitudinal follow-up, alongside either in-hospital birth registration or complete follow-up documentation. Exclusion criteria comprised: (1) confirmed diagnoses of syndromic vascular anomalies, including but not limited to PHACE syndrome, Sturge-Weber syndrome, or CLOVES syndrome, as well as concurrent non-hemangioma vascular malformations; (2) known chromosomal aberrations or major structural defects such as trisomy 21 or severe cardiac and cerebral malformations; (3) extreme prematurity (gestational age <28 weeks) or extremely low birth weight (<1,000 g); (4) presence of profound immunodeficiency or antecedent neoplastic conditions, including congenital immunodeficiency syndromes; (5) mortality or attrition within 12 months postpartum. In this study, the case group comprised infants and young children with clinically, radiologically, and pathologically confirmed IH. The control group was drawn from contemporaneous infants undergoing routine check-ups or clinical visits at the same hospitals, all of whom were systematically screened to exclude IH, other vascular tumors, and congenital vascular malformations. Furthermore, individuals with evident infections, immunological disorders, metabolic diseases, or other severe systemic conditions were excluded to ensure that the control group accurately represented a population of “healthy infants without IH.” This retrospective investigation received ethical approval from the institutional review boards of all participating centers, with informed consent requirements duly waived.

### Study design and data collection

A comprehensive set of 40 clinical variables encompassing diverse domains was systematically collected to enable an exhaustive risk assessment for infantile hemangiomas. These variables comprised: Demographic and parental factors including infant sex, small-for-gestational-age (SGA) status, parental age, and mode of delivery; Perinatal and obstetric history such as maternal American Society of Anesthesiologists (ASA) score, maternal smoking and alcohol consumption, history of miscarriage, placental abnormalities, paternal smoking and alcohol use, family history of hemangiomas, multiple gestation, prematurity, and low birth weight. Maternal comorbidities and antenatal conditions included hormone therapy during pregnancy, intrauterine infection, gestational hypertension, gestational diabetes, maternal anemia, and umbilical cord complications. Neonatal conditions and congenital anomalies encompassed Apgar scores, congenital heart disease (CHD), and gestational age classification. Laboratory biomarkers incorporated serum albumin (ALB), C-reactive protein (CRP), serum amyloid A (SAA), vascular endothelial growth factor (VEGF), interleukin-6 (IL-6), tumor necrosis factor-alpha (TNF-α), and neutrophil-to-lymphocyte ratio (NLR). Tumor characteristics and clinical manifestations were also recorded, including hemangioma subtype, age at onset, lesion size and morphology, anatomical location, presence of complications, and lesion count. The principal outcome measure was the occurrence of infantile hemangioma. In this study, biomarkers including VEGF, CRP, and SAA were derived from blood tests conducted during routine postnatal check-ups and early clinical visits, collected within 1–6 months after birth. The majority of samples were obtained prior to the clinical confirmation of IH or during the early stage of the lesion, typically at the time when a suspicious lesion was first identified. Recognizing that SAA and CRP are acute-phase reactants susceptible to elevation during acute infections, which could confound study outcomes, we implemented rigorous measures during study design and data collection to mitigate this effect. All enrolled infants underwent comprehensive clinical evaluation at the time of sampling, with those exhibiting overt signs of infection (e.g., fever, respiratory or urinary tract infections) systematically excluded. Beyond SAA and CRP, additional infection-related laboratory indices, including white blood cell count and neutrophil proportion, were incorporated to identify and omit cases with potential active infection. Sample collection was further confined to infants without a recent history of acute infection (e.g., within the preceding two weeks) to minimize interference. Collectively, these measures ensured that observed variations in SAA and CRP more faithfully reflected the immune-inflammatory milieu pertinent to infantile hemangioma.

### Missing data handling

Variables exhibiting a missing rate below 5% were designated as having low missingness, whereas those with missing rates ranging from 5% to 30% were considered to possess moderate to high missingness. Two complementary strategies were employed to address these gaps. For variables with low missingness, simple imputation was implemented: continuous variables were imputed using the median, and categorical variables were imputed using the mode (most frequent category). This method, confined to scenarios of minimal missingness, aimed to preserve sample integrity and was subsequently benchmarked against multiple imputation outcomes in sensitivity analyses. For variables with moderate to high missingness, multiple imputation was undertaken. Binary variables were imputed via logistic regression models, wherein the probability distribution of missing values was inferred from available predictors, followed by stochastic sampling to retain intrinsic inter-variable correlations. Multicategorical variables were addressed using multinomial logistic regression, concurrently estimating the probability of each mutually exclusive category and imputing missing entries through probabilistic sampling. This approach preserved the original data distribution, mitigated bias, enhanced the plausibility of imputations, and consequently bolstered the predictive robustness of downstream models.

### Diagnosis of infantile hemangioma and definition of associated factors

The diagnosis of infantile hemangioma was ascertained through a comprehensive clinical framework, predominantly grounded in meticulous physical examination and augmented by high-resolution imaging modalities as warranted (18–20). Initial assessments were performed by seasoned pediatricians or dermatologists, drawing upon key clinical hallmarks including lesion onset, rapid proliferative behavior, characteristic coloration ranging from vivid red to bluish-purple, soft and elevated consistency, blanching response, and the quintessential triphasic pattern of proliferation, plateau, and involution.

For lesions exhibiting classical phenotypes, diagnosis was rendered on clinical grounds alone. In instances of atypical morphology, suspected deep tissue infiltration, or to discriminate from alternative vascular anomalies such as vascular malformations or angiosarcoma, color Doppler ultrasonography was employed to appraise lesion depth, margins, internal structure, and hemodynamic features. Lesions located in anatomically intricate regions—such as the orbit, cervical area, oropharynx, or visceral organs—or those demonstrating extensive involvement, were further evaluated using magnetic resonance imaging (MRI) to precisely delineate lesion boundaries and their spatial relationships with adjacent tissues. Cases presenting equivocal imaging findings underwent multidisciplinary review by senior radiologists. Definitive diagnoses were established through consensus by no fewer than two senior pediatric specialists, synthesizing clinical presentation, imaging data, and longitudinal follow-up, thereby ensuring strict adherence to diagnostic criteria for infantile hemangioma among all enrolled subjects.

### Development and evaluation of predictive models for machine learning algorithms

This study utilized SPSS and R software to develop and systematically evaluate a clinical prediction model through the following steps:

1. Data preprocessing.

The study population consisted of infants and young children treated at Wuxi People's Hospital and Wuxi Second People's Hospital from January 2020 to January 2024, designated as the internal validation cohort. Concurrently, patients with comparable conditions from Tengzhou Central People's Hospital during the same period formed the external validation cohort to assess model generalizability. Within the internal cohort, stratified random sampling was applied to split the data into training and test sets at a 7:3 ratio. This stratification aimed to enhance detection of low-prevalence outcomes, such as infantile hemangioma, mitigating bias toward the majority class and improving both predictive performance and clinical applicability.

2. Variable selection.

A systematic statistical analysis of candidate variables within the internal cohort was performed to identify clinical characteristics significantly associated with infantile hemangioma. Univariate analyses employed chi-square tests for categorical variables and independent-samples t-tests for continuous variables, with variables reaching significance (*P* < 0.05) considered potential risk factors. These variables were further analyzed in a multivariable logistic regression model to control for confounding and identify independent predictors, with adjusted odds ratios and 95% confidence intervals quantifying their predictive strength. Complementing traditional statistical approaches, four classical machine learning algorithms—Extreme Gradient Boosting (XGBoost), Random Forest (RF), Support Vector Machine (SVM), and k-Nearest Neighbor (KNN)—were applied to assess feature importance in a high-dimensional context. Cross-validation of feature importance rankings across the four models identified the top ten consistently ranked features, which were selected as key predictors. This consensus-driven, multi-algorithm feature selection strategy enhanced robustness and interpretability, ensuring consistency across modeling frameworks and providing a solid foundation for model development. In this study, hyperparameter optimization for all four models was conducted via grid search, systematically exploring every possible combination within a predefined parameter space and assessing model performance through cross-validation to identify the configuration that maximized validation set outcomes. This exhaustive strategy ensures that potentially optimal parameter sets are not overlooked and is especially suited to moderately sized search spaces. Despite its computational demands, grid search offers robust stability and reliability in hyperparameter selection, thereby enhancing model generalizability and predictive precision. Coupled with ten-fold cross-validation, this approach effectively mitigates overfitting and upholds the rigor and scientific integrity of the tuning process.

3. Model construction and evaluation.

The selected features were incorporated into the four machine learning models to predict infantile hemangioma risk. Model performance was assessed across three dimensions: discrimination, calibration, and clinical utility. Discriminative ability was evaluated via receiver operating characteristic (ROC) curves and area under the curve (AUC). Calibration was assessed with calibration plots comparing predicted vs. observed outcomes, supplemented by Brier scores as quantitative measures. Clinical utility was evaluated using decision curve analysis (DCA), wherein the *x*-axis represents threshold probability—reflecting the risk level at which clinical intervention is warranted—and the *y*-axis denotes net benefit, balancing true positive gains against overtreatment harms. DCA includes three reference curves: model prediction, treat-all, and treat-none strategies. Superior clinical value is indicated when the model's net benefit curve surpasses the extremes. To improve generalizability and reduce bias from data partitioning, 10-fold cross-validation was implemented. The internal dataset was randomly divided into ten equal, non-overlapping folds; in each iteration, one fold served as validation, while the other nine were combined for training and hyperparameter tuning. Performance metrics including accuracy, AUC, and Brier score were computed per fold and averaged to yield robust estimates, minimizing chance effects and enhancing evaluation reliability.

4. External validation.

The optimal model and hyperparameters identified during internal validation were applied directly to the external cohort. Model performance metrics were recalculated to verify consistency with internal results and evaluate generalizability and clinical utility in a real-world setting.

5. Assessment of model robustness and performance.

To evaluate the robustness and performance of the model within the constraints of a limited sample size, we conducted a series of *post-hoc* analyses. First, Kolmogorov–Smirnov (KS) curves were constructed to assess the separation of predicted risk scores between IH cases and non-IH controls. Second, confusion matrices for both the training and testing sets were generated to visually appraise classification performance, delineating true positives (TP), false negatives (FN), false positives (FP), and true negatives (TN), thereby offering a precise representation of the model's capacity to accurately discriminate IH from non-IH samples. Third, parallel coordinates plots were employed to interrogate the contribution of individual features to predictions and to evaluate the consistency of prediction patterns across samples. Collectively, these analyses demonstrate that, notwithstanding the limited IH case count, the model sustained stable discriminative power and consistent predictive behavior, corroborating the reliability of the study's findings.

6. Model interpretation.

To elucidate model decision mechanisms, SHapley Additive exPlanations (SHAP) were utilized. SHAP values quantify each feature's marginal contribution across varying feature combinations, providing a fair attribution of variable impact on overall predictions. SHAP visualizations enhanced transparency and interpretability: summary plots displayed distributions of SHAP values for all features across samples, indicating feature importance and effect directionality, with each dot representing a sample's SHAP value colored by original feature value. This visualization identified dominant risk factors and their impact patterns. Additionally, SHAP force plots offered individualized explanations, illustrating how each feature influenced a single sample's prediction through positive or negative “forces,” beginning from a baseline and culminating in the predicted risk. These plots facilitate interpretation at both population and individual levels, supporting personalized risk profiling.

## Results

### Basic clinical information of the patient

**Figure 1:**
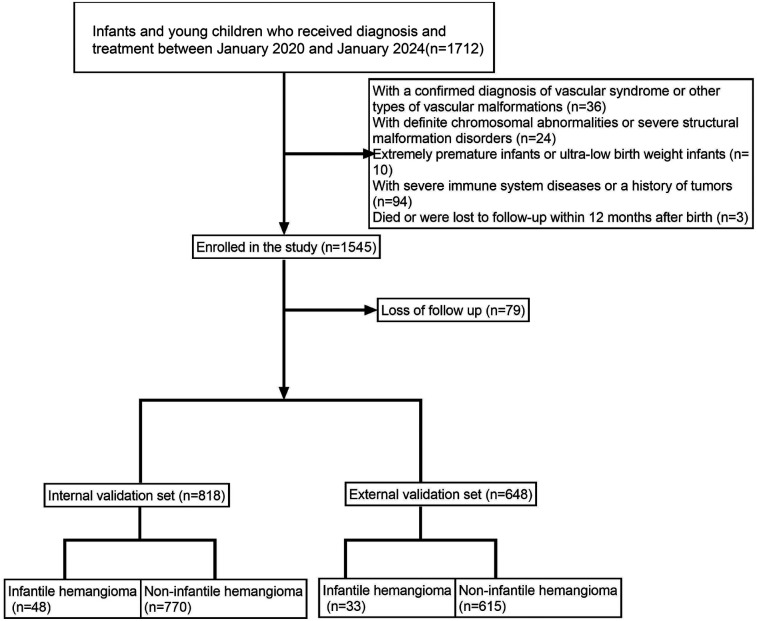
Illustrates the patient enrollment flowchart, clearly outlining the sample selection process.

A total of 1,466 infants and young children were enrolled in the study, including 81 cases of hemangioma (Table 1, Supplementary Table S1 and Figure 1). The cohort was predominantly female (71.6%), with males comprising 28.4%. The median age at onset was 18.0 months. Within the group, 33.3% were SGA, 27.2% were firstborns, and 35.8% had a neonatal Apgar score below 7. Multiple gestations accounted for 53.1% of cases, 44.4% were born prematurely, and 60.5% had low birth weight. Nuchal cord occurrence was observed in 16.0% of neonates. Consistent with prior research, hemangiomas were classified morphologically into focal, segmental, indeterminate, and multifocal subtypes. Focal hemangiomas predominated, representing 61.7% of cases, followed by indeterminate (24.7%) and segmental (12.3%) types; multifocal lesions were rare, present in only 1.2% of patients. The majority of children (77.8%) presented with solitary lesions, whereas 22.2% exhibited multiple lesions. Lesion distribution was highest on the head and neck (62.96%), followed by the face (16.0%), trunk (11.1%), extremities (7.4%), and perineum (2.5%). Regarding complications, 65.4% of patients were complication-free. Among those affected, ulceration was the most frequent (22.2%), followed by auditory or airway obstruction (4.9%), vision impairment (2.5%), secondary infection (2.5%), and bleeding (2.5%). Notably, these complications frequently co-occurred with ulceration. The internal dataset included 818 participants, of whom 48 had infantile hemangioma (IH). The external dataset comprised 648 participants, including 33 IH cases. A comparison of the features is presented in Table 2. The internal dataset was randomly divided into training and testing sets at a 7:3 ratio, with their characteristics compared in Table 3. The original dataset utilized in this study is provided in Supplementary Table S2. To ensure the reproducibility and transparency of this study, all source code used—including scripts for data preprocessing, model construction, performance evaluation, and SHAP analysis—is available at the permanent access link (https://www.jianguoyun.com/p/DWh9chMQl-GKDBjEj-sFIAA).

**Table 1:** Provides a detailed overview of the demographic and clinical characteristics of pediatric patients diagnosed with infantile hemangioma.

Characteristic		Hemangioma patients (*N* = 81)
Sex, *N* (%)	Female	58 (71.605%)
	Male	23 (28.395%)
Ageatonset, median [Q1–Q3]	18.000 [9.000;26.000]	
SGA, *N* (%)	No	54 (66.667%)
	Yes	27 (33.333%)
Primiparity, *N* (%)	No	59 (72.840%)
	Yes	22 (27.160%)
Apgar score, *N* (%)	≥7	52 (64.198%)
	<7	29 (35.802%)
MP, *N* (%)	No	38 (46.914%)
	Yes	43 (53.086%)
Preterm birth, *N* (%):	No	45 (55.556%)
	Yes	36 (44.444%)
Low birth weight infant, *N* (%)	No	32 (39.506%)
	Yes	49 (60.494%)
Mode of delivery, *N* (%)	Vaginal delivery	
	Cesarean section	
Nuchal cord, *N* (%)	No	68 (83.951%)
	Yes	13 (16.049%)
Lesion size, median [Q1–Q3]	6.900 [4.200;11.200]	
Lesion type, *N* (%)	Localized	50 (61.728%)
	Segmental	10 (12.346%)
	Indeterminate	20 (24.691%)
	Multifocal	1 (1.235%)
Number of lesions, *N* (%)	Single	63 (77.778%)
	Multiple	18 (22.222%)
Localization, *N* (%)	Head and neck	51 (62.963%)
	Face	13 (16.049%)
	Trunk	9 (11.111%)
	Extremities	6 (7.407%)
	Perineum	2 (2.469%)
Lesions with complications, *N* (%)	Ulceration	18 (22.222%)
	Auditory canal or airway obstruction	4 (4.938%)
	Visual threat or impairment	2 (2.469%)
	Ulceration with secondary infection	2 (2.469%)
	Ulceration with bleeding	2 (2.469%)
	No complications	53 (65.432%)

SGA, small for gestational age; MP, multiple pregnancy*.*

**Table 2:** Comparison of features between the internal and external datasets.

Variables		All (*N* = 1,466)	Internal dataset (*N* = 818)	External dataset (*N* = 648)	*P*-value
Sex	Female	1,014 (69.168%)	561 (68.582%)	453 (69.907%)	0.625
	Male	452 (30.832%)	257 (31.418%)	195 (30.093%)	
Age	<35	1,172 (79.945%)	645 (78.851%)	527 (81.327%)	0.267
	≥35	294 (20.055%)	173 (21.149%)	121 (18.673%)	
Age*	<35	986 (67.258%)	557 (68.093%)	429 (66.204%)	0.478
	≥35	480 (32.742%)	261 (31.907%)	219 (33.796%)	
ASA	<3	1,322 (90.177%)	739 (90.342%)	583 (89.969%)	0.881
	≥3	144 (9.823%)	79 (9.658%)	65 (10.031%)	
Drinking history	No	1,128 (76.944%)	621 (75.917%)	507 (78.241%)	0.324
	Yes	338 (23.056%)	197 (24.083%)	141 (21.759%)	
Smoking history	No	1,322 (90.177%)	729 (89.120%)	593 (91.512%)	0.15
	Yes	144 (9.823%)	89 (10.880%)	55 (8.488%)	
Drinking history*	No	1,016 (69.304%)	547 (66.870%)	469 (72.377%)	0.027
	Yes	450 (30.696%)	271 (33.130%)	179 (27.623%)	
Smoking history*	No	1,226 (83.629%)	679 (83.007%)	547 (84.414%)	0.515
	Yes	240 (16.371%)	139 (16.993%)	101 (15.586%)	
Family history	No	1,272 (86.767%)	697 (85.208%)	575 (88.735%)	0.057
	Yes	194 (13.233%)	121 (14.792%)	73 (11.265%)	
History of miscarriage	No	1,189 (81.105%)	656 (80.196%)	533 (82.253%)	0.351
	Yes	277 (18.895%)	162 (19.804%)	115 (17.747%)	
Primiparity	No	1,220 (83.220%)	660 (80.685%)	560 (86.420%)	0.004
	Yes	246 (16.780%)	158 (19.315%)	88 (13.580%)	
Hormonal therapy	No	1,152 (78.581%)	619 (75.672%)	533 (82.253%)	0.003
	Yes	314 (21.419%)	199 (24.328%)	115 (17.747%)	
Anemia	No	1,346 (91.814%)	744 (90.954%)	602 (92.901%)	0.209
	Yes	120 (8.186%)	74 (9.046%)	46 (7.099%)	
CHD	No	1,258 (85.812%)	676 (82.641%)	582 (89.815%)	<0.001
	Yes	208 (14.188%)	142 (17.359%)	66 (10.185%)	
Hyperlipidemia	No	1,048 (71.487%)	567 (69.315%)	481 (74.228%)	0.044
	Yes	418 (28.513%)	251 (30.685%)	167 (25.772%)	
Infection during pregnancy	No	1,124 (76.671%)	604 (73.839%)	520 (80.247%)	0.005
	Yes	342 (23.329%)	214 (26.161%)	128 (19.753%)	
Gestational hypertension	No	1,048 (71.487%)	559 (68.337%)	489 (75.463%)	0.003
	Yes	418 (28.513%)	259 (31.663%)	159 (24.537%)	
Gestational diabetes mellitus	No	1,010 (68.895%)	535 (65.403%)	475 (73.302%)	0.001
	Yes	456 (31.105%)	283 (34.597%)	173 (26.698%)	
ALB	≥30 g/L	1,146 (78.172%)	617 (75.428%)	529 (81.636%)	0.005
	<30 g/L	320 (21.828%)	201 (24.572%)	119 (18.364%)	
Mode of delivery	Vaginal delivery	1,213 (82.742%)	671 (82.029%)	542 (83.642%)	0.458
	Cesarean section	253 (17.258%)	147 (17.971%)	106 (16.358%)	
Multiple pregnancy	No	1,162 (79.263%)	644 (78.729%)	518 (79.938%)	0.615
	Yes	304 (20.737%)	174 (21.271%)	130 (20.062%)	
Preterm birth	No	1,291 (88.063%)	719 (87.897%)	572 (88.272%)	0.89
	Yes	175 (11.937%)	99 (12.103%)	76 (11.728%)	
Placental abnormalities	No	1,445 (98.568%)	807 (98.655%)	638 (98.457%)	0.923
	Yes	21 (1.432%)	11 (1.345%)	10 (1.543%)	
Nuchal cord	No	1,317 (89.836%)	728 (88.998%)	589 (90.895%)	0.268
	Yes	149 (10.164%)	90 (11.002%)	59 (9.105%)	
Low birth weight infant	No	1,310 (89.359%)	729 (89.120%)	581 (89.660%)	0.804
	Yes	156 (10.641%)	89 (10.880%)	67 (10.340%)	
SGA	No	1,200 (81.855%)	668 (81.663%)	532 (82.099%)	0.883
	Yes	266 (18.145%)	150 (18.337%)	116 (17.901%)	
Apgar score	≥7	1,306 (89.086%)	722 (88.264%)	584 (90.123%)	0.294
	<7	160 (10.914%)	96 (11.736%)	64 (9.877%)	
VEGF level	<115 pg/mL	1,189 (81.105%)	639 (78.117%)	550 (84.877%)	0.001
	≥115 pg/mL	277 (18.895%)	179 (21.883%)	98 (15.123%)	
CRP level	<10 mg/L	1,062 (72.442%)	570 (69.682%)	492 (75.926%)	0.009
	≥10 mg/L	404 (27.558%)	248 (30.318%)	156 (24.074%)	
SAA level	<10 mg/L	1,178 (80.355%)	629 (76.895%)	549 (84.722%)	<0.001
	≥10 mg/L	288 (19.645%)	189 (23.105%)	99 (15.278%)	
NLR	<3	1,110 (75.716%)	597 (72.983%)	513 (79.167%)	0.007
	≥3	356 (24.284%)	221 (27.017%)	135 (20.833%)	
TNF-α	<8 pg/mL	1,174 (80.082%)	637 (77.873%)	537 (82.870%)	0.021
	≥8 pg/mL	292 (19.918%)	181 (22.127%)	111 (17.130%)	
IL-6	<7 pg/mL	1,225 (83.561%)	672 (82.152%)	553 (85.340%)	0.118
	≥7 pg/mL	241 (16.439%)	146 (17.848%)	95 (14.660%)	
Hemangioma	No	1,385 (94.475%)	770 (94.132%)	615 (94.907%)	0.596
	Yes	81 (5.525%)	48 (5.868%)	33 (5.093%)	

Sex, Family history, Primiparity, Mode of delivery, Multiple pregnancy, Preterm birth, Placental abnormalities, Nuchal cord, Low birth weight infant, SGA, Apgar score, VEGF level, CRP level, SAA level, NLR, TNF-α, IL-6 — Infant clinical characteristics; Age, ASA, Drinking history, Smoking history, History of miscarriage, Hormonal therapy, Anemia, CHD, Hyperlipidemia, Infection during pregnancy, Gestational hypertension, Gestational diabetes, ALB — Maternal clinical characteristics; Age*, Drinking history*, Smoking history* — Paternal clinical characteristics. SGA, Small for Gestational Age; VEGF, Vascular Endothelial Growth Factor; CRP, C-Reactive Protein; SAA, Serum Amyloid A; NLR, Neutrophil-to-Lymphocyte Ratio; TNF-α, Tumor Necrosis Factor Alpha; IL-6, Interleukin 6; ASA, Acute Phase Serum Amyloid A; OR, odds ratio; CI, confidence interval.

**Table 3:** Comparison of features between the training and testing datasets.

Variables		All (*N* = 818)	Training set (*N* = 572)	Testing set (*N* = 246)	*P*-value
Sex	Female	561 (68.582%)	401 (70.105%)	160 (65.041%)	0.177
	Male	257 (31.418%)	171 (29.895%)	86 (34.959%)	
Age	<35	645 (78.851%)	447 (78.147%)	198 (80.488%)	0.51
	≥35	173 (21.149%)	125 (21.853%)	48 (19.512%)	
Age*	<35	557 (68.093%)	392 (68.531%)	165 (67.073%)	0.742
	≥35	261 (31.907%)	180 (31.469%)	81 (32.927%)	
ASA	<3	739 (90.342%)	516 (90.210%)	223 (90.650%)	0.947
	≥3	79 (9.658%)	56 (9.790%)	23 (9.350%)	
Drinking history	No	621 (75.917%)	432 (75.524%)	189 (76.829%)	0.756
	Yes	197 (24.083%)	140 (24.476%)	57 (23.171%)	
Smoking history	No	729 (89.120%)	512 (89.510%)	217 (88.211%)	0.671
	Yes	89 (10.880%)	60 (10.490%)	29 (11.789%)	
Drinking history*	No	547 (66.870%)	382 (66.783%)	165 (67.073%)	1
	Yes	271 (33.130%)	190 (33.217%)	81 (32.927%)	
Smoking history*	No	679 (83.007%)	475 (83.042%)	204 (82.927%)	1
	Yes	139 (16.993%)	97 (16.958%)	42 (17.073%)	
Family history	No	697 (85.208%)	486 (84.965%)	211 (85.772%)	0.849
	Yes	121 (14.792%)	86 (15.035%)	35 (14.228%)	
History of miscarriage	No	656 (80.196%)	454 (79.371%)	202 (82.114%)	0.42
	Yes	162 (19.804%)	118 (20.629%)	44 (17.886%)	
First pregnancy	No	660 (80.685%)	466 (81.469%)	194 (78.862%)	0.442
	Yes	158 (19.315%)	106 (18.531%)	52 (21.138%)	
Hormonal therapy	No	619 (75.672%)	441 (77.098%)	178 (72.358%)	0.174
	Yes	199 (24.328%)	131 (22.902%)	68 (27.642%)	
Anemia	No	744 (90.954%)	523 (91.434%)	221 (89.837%)	0.551
	Yes	74 (9.046%)	49 (8.566%)	25 (10.163%)	
CHD	No	676 (82.641%)	474 (82.867%)	202 (82.114%)	0.873
	Yes	142 (17.359%)	98 (17.133%)	44 (17.886%)	
Hyperlipidemia	No	567 (69.315%)	391 (68.357%)	176 (71.545%)	0.41
	Yes	251 (30.685%)	181 (31.643%)	70 (28.455%)	
Infection during pregnancy	No	604 (73.839%)	425 (74.301%)	179 (72.764%)	0.71
	Yes	214 (26.161%)	147 (25.699%)	67 (27.236%)	
Gestational hypertension	No	559 (68.337%)	385 (67.308%)	174 (70.732%)	0.377
	Yes	259 (31.663%)	187 (32.692%)	72 (29.268%)	
Gestational diabetes mellitus	No	535 (65.403%)	393 (68.706%)	142 (57.724%)	0.003
	Yes	283 (34.597%)	179 (31.294%)	104 (42.276%)	
ALB	≥30 g/L	617 (75.428%)	434 (75.874%)	183 (74.390%)	0.716
	<30 g/L	201 (24.572%)	138 (24.126%)	63 (25.610%)	
Mode of delivery	Vaginal delivery	671 (82.029%)	480 (83.916%)	191 (77.642%)	0.041
	Cesarean section	147 (17.971%)	92 (16.084%)	55 (22.358%)	
Multiple pregnancy	No	644 (78.729%)	448 (78.322%)	196 (79.675%)	0.733
	Yes	174 (21.271%)	124 (21.678%)	50 (20.325%)	
Preterm birth	No	719 (87.897%)	497 (86.888%)	222 (90.244%)	0.218
	Yes	99 (12.103%)	75 (13.112%)	24 (9.756%)	
Placental abnormalities	No	807 (98.655%)	566 (98.951%)	241 (97.967%)	0.321
	Yes	11 (1.345%)	6 (1.049%)	5 (2.033%)	
Nuchal cord	No	728 (88.998%)	505 (88.287%)	223 (90.650%)	0.385
	Yes	90 (11.002%)	67 (11.713%)	23 (9.350%)	
Low birth weight infant	No	729 (89.120%)	507 (88.636%)	222 (90.244%)	0.579
	Yes	89 (10.880%)	65 (11.364%)	24 (9.756%)	
SGA	No	668 (81.663%)	479 (83.741%)	189 (76.829%)	0.025
	Yes	150 (18.337%)	93 (16.259%)	57 (23.171%)	
Apgar score	≥7	722 (88.264%)	512 (89.510%)	210 (85.366%)	0.116
	<7	96 (11.736%)	60 (10.490%)	36 (14.634%)	
VEGF level	<115 pg/mL	639 (78.117%)	441 (77.098%)	198 (80.488%)	0.326
	≥115 pg/mL	179 (21.883%)	131 (22.902%)	48 (19.512%)	
CRP level	<10 mg/L	570 (69.682%)	393 (68.706%)	177 (71.951%)	0.399
	≥10 mg/L	248 (30.318%)	179 (31.294%)	69 (28.049%)	
SAA level	<10 mg/L	629 (76.895%)	443 (77.448%)	186 (75.610%)	0.63
	≥10 mg/L	189 (23.105%)	129 (22.552%)	60 (24.390%)	
NLR	<3	597 (72.983%)	415 (72.552%)	182 (73.984%)	0.736
	≥3	221 (27.017%)	157 (27.448%)	64 (26.016%)	
TNF-α	<8 pg/mL	637 (77.873%)	454 (79.371%)	183 (74.390%)	0.138
	≥8 pg/mL	181 (22.127%)	118 (20.629%)	63 (25.610%)	
IL-6	<7 pg/mL	672 (82.152%)	476 (83.217%)	196 (79.675%)	0.265
	≥7 pg/mL	146 (17.848%)	96 (16.783%)	50 (20.325%)	
Hemangioma	No	770 (94.132%)	537 (93.881%)	233 (94.715%)	0.762
	Yes	48 (5.868%)	35 (6.119%)	13 (5.285%)	

Sex, Family history, Primiparity, Mode of delivery, Multiple pregnancy, Preterm birth, Placental abnormalities, Nuchal cord, Low birth weight infant, SGA, Apgar score, VEGF level, CRP level, SAA level, NLR, TNF-α, IL-6 — Infant clinical characteristics; Age, ASA, Drinking history, Smoking history, History of miscarriage, Hormonal therapy, Anemia, CHD, Hyperlipidemia, Infection during pregnancy, Gestational hypertension, Gestational diabetes, ALB — Maternal clinical characteristics; Age*, Drinking history*, Smoking history* — Paternal clinical characteristics. SGA, Small for Gestational Age; VEGF, Vascular Endothelial Growth Factor; CRP, C-Reactive Protein; SAA, Serum Amyloid A; NLR, Neutrophil-to-Lymphocyte Ratio; TNF-α, Tumor Necrosis Factor Alpha; IL-6, Interleukin 6; ASA, Acute Phase Serum Amyloid A; OR, odds ratio; CI, confidence interval.

### Identification of risk factors for infantile hemangioma

Both univariate and multivariate logistic regression analyses identified several independent risk factors for infantile hemangioma development, including gestational diabetes mellitus, mode of delivery, multiple pregnancy, preterm birth, low birth weight, Apgar score, and elevated levels of VEGF, CRP, and SAA (*P* < 0.05) (Table 4). These results highlight the multifactorial etiology of infantile hemangioma, implicating perinatal factors alongside inflammatory and angiogenic biomarkers in its pathogenesis.

**Table 4:** Summarizes the findings from univariate and multivariate analyses identifying variables significantly associated with infantile hemangioma.

Variables		*N*	Univariate analysis			Multivariate analysis		
			OR	95%CI	*P*-value	OR	95%CI	*P*-value
Sex	Female	561	Reference					
	Male	257	0.992	[0.529,1.860]	0.979			
Age	<35	645	Reference			Reference		
	≥35	173	6.034	[3.307,11.013]	<0.001	2.663	[0.930, 7.563]	0.065
Age*	<35	557	Reference			Reference		
	≥35	261	1.88	[1.044,3.384]	0.035	2.422	[0.850, 7.056]	0.098
ASA	<3	739	Reference			Reference		
	≥3	79	2.674	[1.277,5.599]	0.009	2.126	[0.575, 7.126]	0.235
Drinking history	No	621	Reference					
	Yes	197	1.321	[0.694,2.515]	0.397			
Smoking history	No	729	Reference					
	Yes	89	0.95	[0.366,2.464]	0.915			
Drinking history*	No	547	Reference			Reference		
	Yes	271	4.444	[2.392,8.254]	<0.001	1.643	[0.593, 4.607]	0.338
Smoking history*	No	679	Reference					
	Yes	139	0.684	[0.285,1.642]	0.396			
Family history	No	697	Reference					
	Yes	121	1.356	[0.639,2.876]	0.428			
History of miscarriage	No	656	Reference					
	Yes	162	1.731	[0.905,3.307]	0.097			
Primiparity	No	660	Reference					
	Yes	158	1.601	[0.826,3.103]	0.163			
Hormonal therapy	No	619	Reference					
	Yes	199	0.809	[0.395,1.655]	0.562			
Anemia	No	744	Reference					
	Yes	74	1.791	[0.774,4.149]	0.174			
CHD	No	676	Reference					
	Yes	142	1.272	[0.618,2.617]	0.513			
Hyperlipidemia	No	567	Reference					
	Yes	251	0.926	[0.488,1.758]	0.814			
Infection during pregnancy	No	604	Reference			Reference		
	Yes	214	0.386	[0.162,0.921]	0.032	0.302	[0.067, 1.084]	0.089
Gestational hypertension	No	559	Reference					
	Yes	259	1.447	[0.795,2.632]	0.226			
Gestational diabetes mellitus	No	535	Reference			Reference		
	Yes	283	3.101	[1.706,5.637]	<0.001	2.919	[1.149, 7.839]	0.027
ALB	≥30 g/L	617	Reference					
	<30 g/L	201	1.15	[0.596,2.219]	0.677			
Mode of delivery	Vaginal delivery	671	Reference			Reference		
	Cesarean section	147	2.7	[1.451,5.023]	0.002	4.41	[1.225, 16.865]	0.025
Multiple pregnancy	No	644	Reference			Reference		
	Yes	174	6.579	[3.589,12.060]	<0.001	5.139	[1.910, 14.758]	0.002
Preterm birth	No	719	Reference			Reference		
	Yes	99	7.615	[4.119,14.081]	<0.001	3.733	[1.170, 11.957]	0.025
Placental abnormalities	No	807	Reference					
	Yes	11	3.676	[0.772,17.509]	0.102			
Nuchal cord	No	728	Reference					
	Yes	90	1.678	[0.759,3.708]	0.201			
Low birth weight infant	No	729	Reference			Reference		
	Yes	89	20.085	[10.571,38.161]	<0.001	32.241	[10.979, 111.198]	<0.001
SGA	No	668	Reference			Reference		
	Yes	150	2.626	[1.412,4.884]	0.002	0.735	[0.194, 2.592]	0.638
Apgar score	≥7	722	Reference			Reference		
	<7	96	4.312	[2.267,8.205]	<0.001	4.317	[1.217, 15.073]	0.022
VEGF level	<115 pg/mL	639	Reference			Reference		
	≥115 pg/mL	179	4.766	[2.629,8.638]	<0.001	9.105	[3.086, 29.906]	<0.001
CRP level	<10 mg/L	570	Reference			Reference		
	≥10 mg/L	248	2.917	[1.619,5.256]	<0.001	8.898	[3.206, 27.778]	<0.001
SAA level	<10 mg/L	629	Reference			Reference		
	≥10 mg/L	189	10.769	[5.563,20.849]	<0.001	6.126	[2.222, 18.096]	0.001
NLR	<3	597	Reference					
	≥3	221	1.004	[0.521,1.934]	0.992			
TNF-α	<8 pg/mL	637	Reference			Reference		
	≥8 pg/mL	181	2.026	[1.094,3.752]	0.025	1.091	[0.357, 3.123]	0.874
IL-6	<7 pg/mL	672	Reference					
	≥7 pg/mL	146	1.227	[0.597,2.522]	0.578			

Sex, Family history, Primiparity, Mode of delivery, Multiple pregnancy, Preterm birth, Placental abnormalities, Nuchal cord, Low birth weight infant, SGA, Apgar score, VEGF level, CRP level, SAA level, NLR, TNF-α, IL-6 — Infant clinical characteristics; Age, ASA, Drinking history, Smoking history, History of miscarriage, Hormonal therapy, Anemia, CHD, Hyperlipidemia, Infection during pregnancy, Gestational hypertension, Gestational diabetes, ALB — Maternal clinical characteristics; Age*, Drinking history*, Smoking history* — Paternal clinical characteristics. SGA, Small for Gestational Age; VEGF, Vascular Endothelial Growth Factor; CRP, C-Reactive Protein; SAA, Serum Amyloid A; NLR, Neutrophil-to-Lymphocyte Ratio; TNF-α, Tumor Necrosis Factor Alpha; IL-6, Interleukin 6; ASA, Acute Phase Serum Amyloid A; OR, odds ratio; CI, confidence interval.

**Figure 2:**
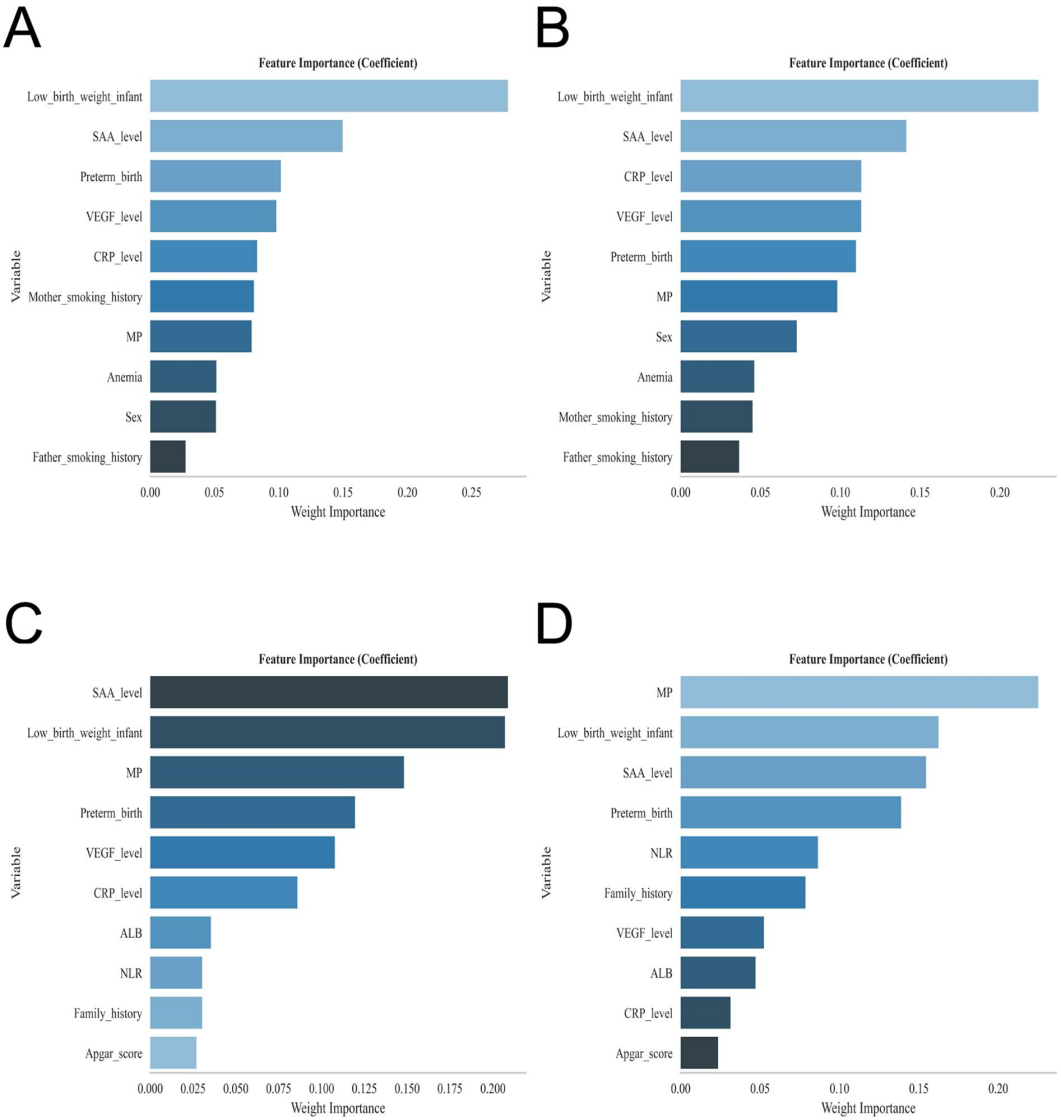
Displays the feature importance rankings for each predictive model: **(A)** extreme gradient boosting (XGBoost), **(B)** random forest (RF), **(C)** support vector machine (SVM), and **(D)** k-nearest neighbor (KNN).

To further refine the risk factor profile, we employed four classical machine learning algorithms—XGBoost, RF, SVM, and KNN—for feature selection. The overlap of top-ranked features across all models consistently identified multiple pregnancy, preterm birth, low birth weight, and elevated VEGF, CRP, and SAA levels as the strongest predictors of infantile hemangioma (Figures 2A–D). This machine learning–augmented strategy corroborated the logistic regression findings and enhanced the robustness of the key predictive variables. The hyperparameters of the four machine learning models were optimized via grid search, with XGBoost set as colsample_bytree = 1, learning_rate = 0.3, max_depth = 4, min_child_weight = 4, n_estimators = 20, reg_lambda = 0.5, and subsample = 1; RF as criterion = gini, max_depth = None, max_features = sqrt, min_impurity_decrease = 0.0, min_samples_leaf = 1, min_samples_split = 2, and n_estimators = 100; SVM as C = 1.0, gamma = scale, kernel = rbf, max_iter = 50, probability = True, and tol = 0.001; and KNN as algorithm = auto, leaf_size = 10, n_neighbors = 4, *p* = 2, and weights = uniform.

### Model building and evaluation

**Figure 3:**
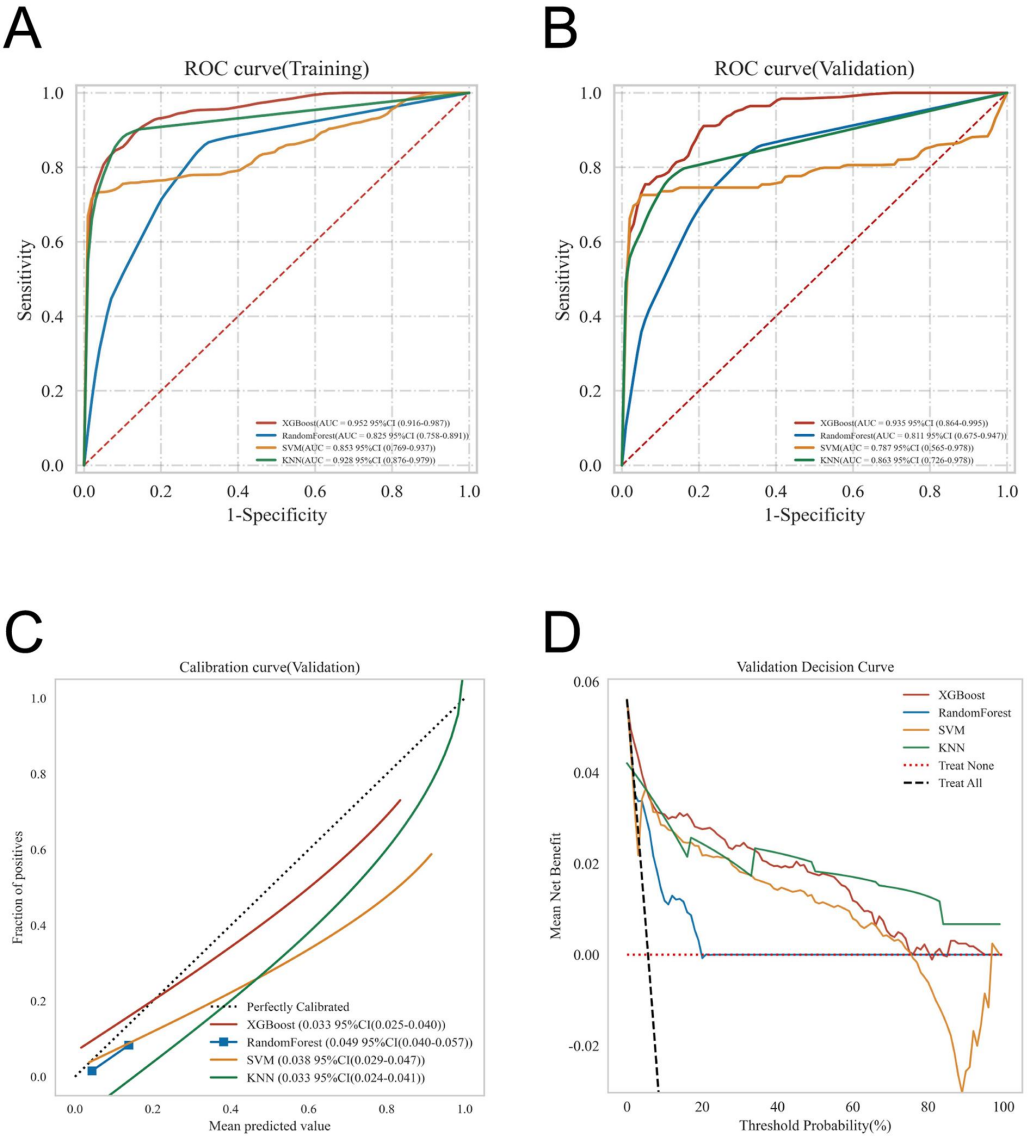
Offers a comprehensive evaluation of the models’ predictive performance, including: **(A)** ROC curves for the training dataset; **(B)** ROC curves for the validation dataset; **(C)** calibration curves, where the 45° dashed line represents perfect alignment between predicted and observed outcomes—curves closer to this line indicate superior calibration; and **(D)** decision curve analysis (DCA), with the red curve illustrating the model's net clinical benefit across varying risk thresholds. The intersections between the red curve and the “All” and “None” strategies define the ranges of risk thresholds where the model provides clinical utility.

**Figure 4:**
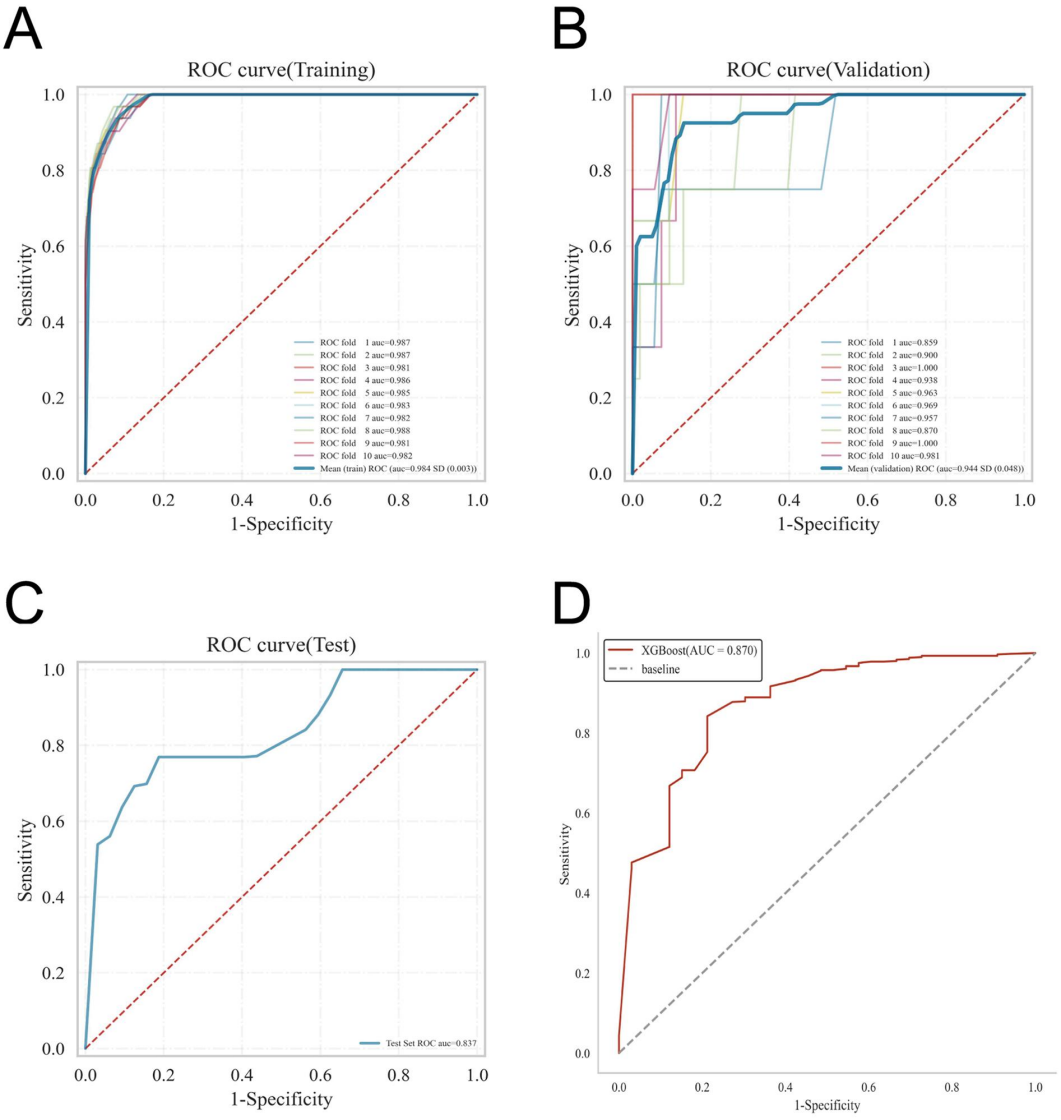
Presents the internal and external validation results for the XGBoost model: **(A)** ROC curve from the training set; **(B)** ROC curve from the validation set; **(C)** ROC curve from the testing set; and **(D)** ROC curve from the external validation cohort.

**Figure 5:**
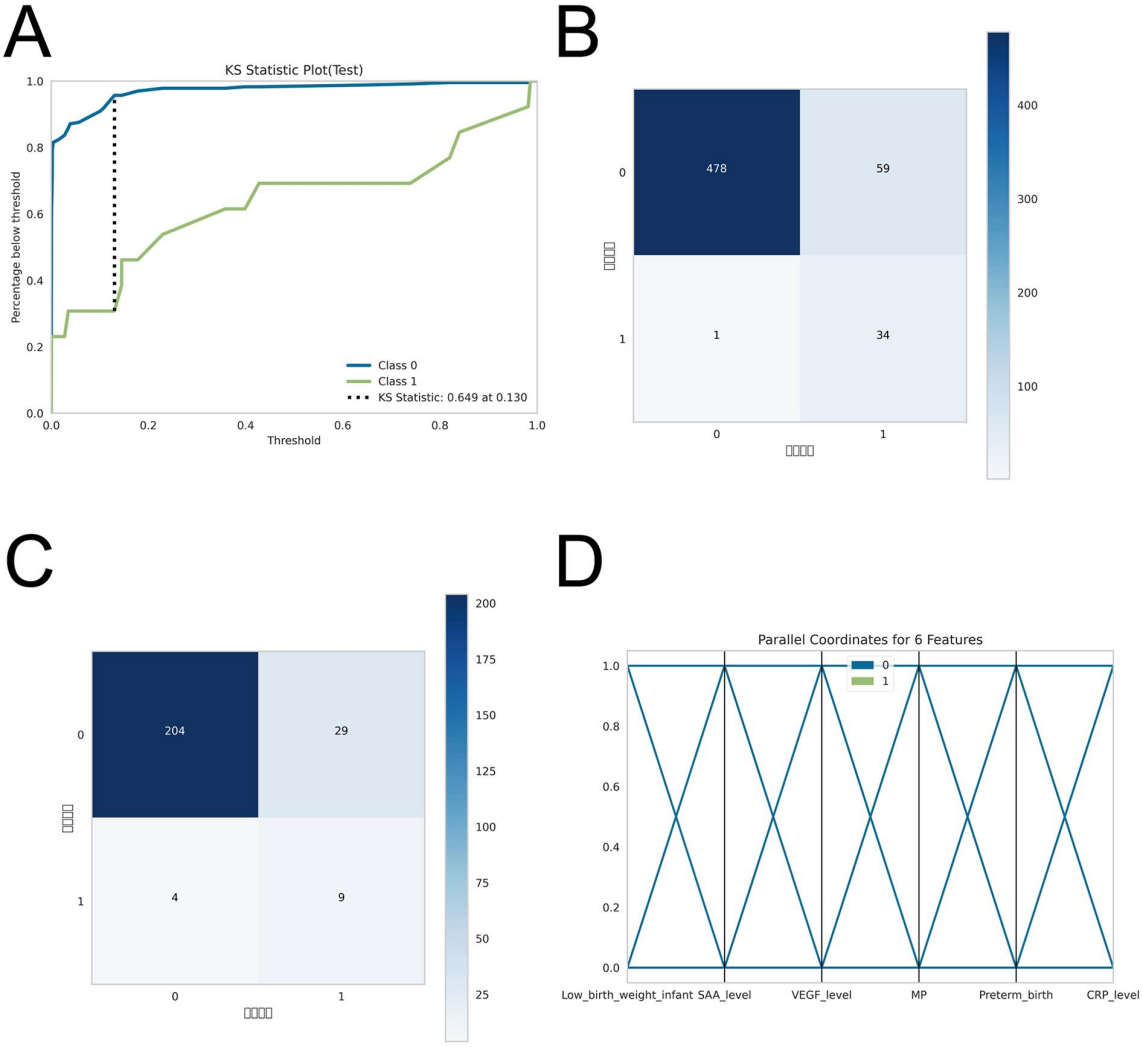
*post-hoc* analyses for model robustness and performance. To evaluate model stability given the limited sample size, **(A)** Kolmogorov–Smirnov (KS) curves were generated to assess separation of predicted risk scores between IH cases and non-IH controls. (B–C) Confusion matrices for the training and testing sets compare predicted outcomes with true labels, showing the numbers of true positives (TP), false negatives (FN), false positives (FP), and true negatives (TN), thereby illustrating the model's classification accuracy. **(D)** Parallel coordinates plots display the contribution of each feature to predictions and assess consistency of prediction patterns across samples.

ROC curve analysis demonstrated that the XGBoost model exhibited superior predictive performance in both the training and validation cohorts, achieving an AUC of 0.952 in the training set and 0.935 in the validation set—the highest among the four evaluated machine learning algorithms (Table 5 and Figures 3A–C). These elevated AUC values underscore the model's excellent discriminative ability to differentiate between high- and low-risk infants, reflecting a high degree of predictive accuracy. Calibration curves for XGBoost, RF, SVM, and KNN revealed strong agreement between predicted probabilities and observed outcomes, indicating good calibration and reliable probability estimation across all models. Additionally, DCA assessed the clinical utility of each model, demonstrating that across a spectrum of threshold probabilities, all models provided greater net clinical benefit compared to “treat-all” or “treat-none” approaches (Figure 3D). Notably, XGBoost delivered the most favorable clinical decision support, highlighting its promise for personalized risk stratification in infantile hemangioma. To rigorously evaluate model generalizability, 10-fold cross-validation was performed within the internal cohort. Specifically, 245 cases (30.0%) were randomly assigned as a test set, while the remainder were used for training and cross-validation. This approach minimized sampling bias and enhanced robustness by averaging performance across multiple data partitions. In cross-validation, XGBoost achieved the highest overall performance with a validation AUC of 0.9438 ± 0.0484, test set AUC of 0.8366, and accuracy of 0.8943 (Figures 4A–C). By comparison, the RF model showed a validation AUC of 0.8510 ± 0.1334, test AUC of 0.8353, and accuracy of 0.8415; SVM yielded a validation AUC of 0.8326 ± 0.1362, test AUC of 0.6827, but the highest accuracy at 0.9472; and KNN demonstrated a validation AUC of 0.8466 ± 0.1243, test AUC of 0.8064, and accuracy of 0.8780. These results collectively emphasize the consistent superiority of XGBoost in terms of AUC, accuracy, and stability, establishing it as the most effective algorithm for predicting high-risk infantile hemangioma. External validation using an independent cohort further corroborated the model's generalizability, with XGBoost achieving an AUC of 0.870 (Figure 4D), confirming robust predictive capability on unseen data. The Kolmogorov–Smirnov (KS) curve demonstrates a clear separation between the cumulative distribution curves of IH cases and non-IH controls, with a pronounced maximum vertical distance (KS value), indicating the model's efficacy in distinguishing high-risk from low-risk samples. The confusion matrices for both the training and testing sets reveal that true positives (TP) and true negatives (TN) markedly exceed false positives (FP) and false negatives (FN), underscoring the model's robust classification performance and accuracy. Parallel coordinates plots exhibit consistent line patterns across samples for different features, effectively illustrating each feature's contribution to model predictions and highlighting distinctions in the multi-feature space between high-risk and low-risk samples, with prediction patterns remaining stable and devoid of notable anomalies (Figures 5A–D).

**Table 5:** Presents the performance metrics of the four predictive models assessed in this study.

Machine learning algorithms	Cohort	AUC (95% CI)	Accuracy (95% CI)	Sensitivity (95% CI)	Specificity (95% CI)	F1 score (95% CI)
KNN	Training set	0.928 (0.876–0.979)	0.899 (0.884–0.914)	0.897 (0.876–0.919)	0.899 (0.884–0.915)	0.519 (0.487–0.552)
	Validation set	0.863 (0.726–0.978)	0.878 (0.863–0.893)	0.783 (0.685–0.880)	0.886 (0.866–0.906)	0.411 (0.356–0.465)
XGBoost	Training set	0.952 (0.916–0.987)	0.898 (0.872–0.925)	0.88 (0.856–0.903)	0.9 (0.870–0.929)	0.524 (0.469–0.580)
	Validation set	0.935 (0.864–0.995)	0.88 (0.852–0.908)	0.743 (0.648–0.839)	0.89 (0.855–0.924)	0.416 (0.356–0.476)
RF	Training set	0.825 (0.758–0.891)	0.718 (0.693–0.744)	0.848 (0.806–0.889)	0.71 (0.681–0.740)	0.265 (0.249–0.282)
	Validation set	0.811 (0.675–0.947)	0.722 (0.681–0.762)	0.836 (0.747–0.925)	0.717 (0.671–0.762)	0.253 (0.212–0.295)
SVM	Training set	0.853 (0.769–0.937)	0.958 (0.943–0.973)	0.74 (0.726–0.754)	0.972 (0.955–0.988)	0.692 (0.634–0.750)
	Validation set	0.787 (0.565–0.978)	0.945 (0.927–0.964)	0.641 (0.527–0.755)	0.965 (0.941–0.988)	0.566(0.499–0.634)

CI, confidence interval.

### Model explanation

**Figure 6:**
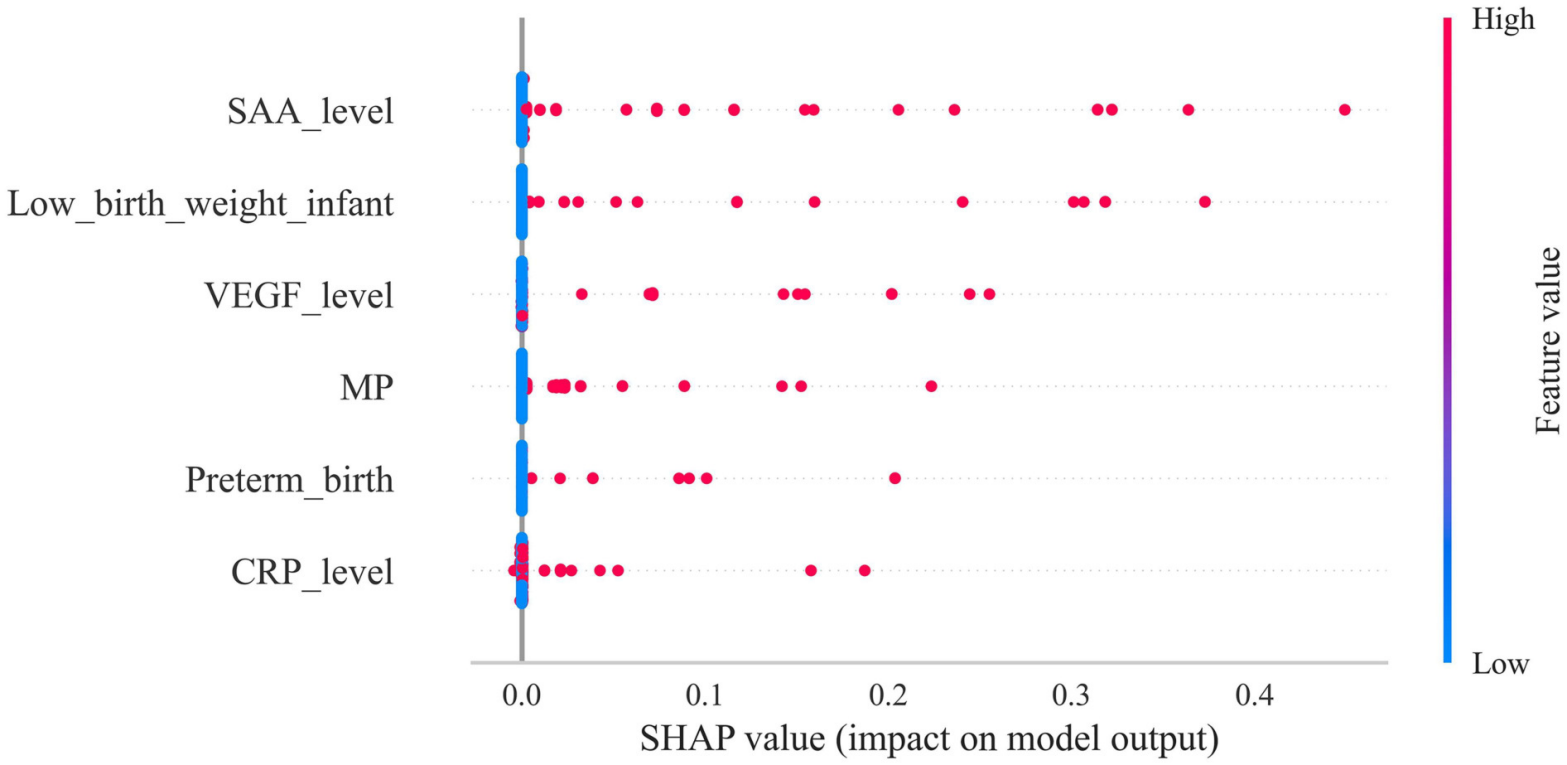
Depicts the SHAP summary plot, ranking risk factors according to their mean absolute shapley values, with higher-ranked factors exerting greater influence on model predictions.

The SHAP summary plot (Figure 6) offers a lucid visualization of the principal risk factors associated with infantile hemangioma, ranking them according to their relative contribution to the model's output. The analysis identified SAA level, low birth weight, VEGF level, multiple pregnancy, preterm birth, and CRP level as the most influential predictors.

**Figure 7:**
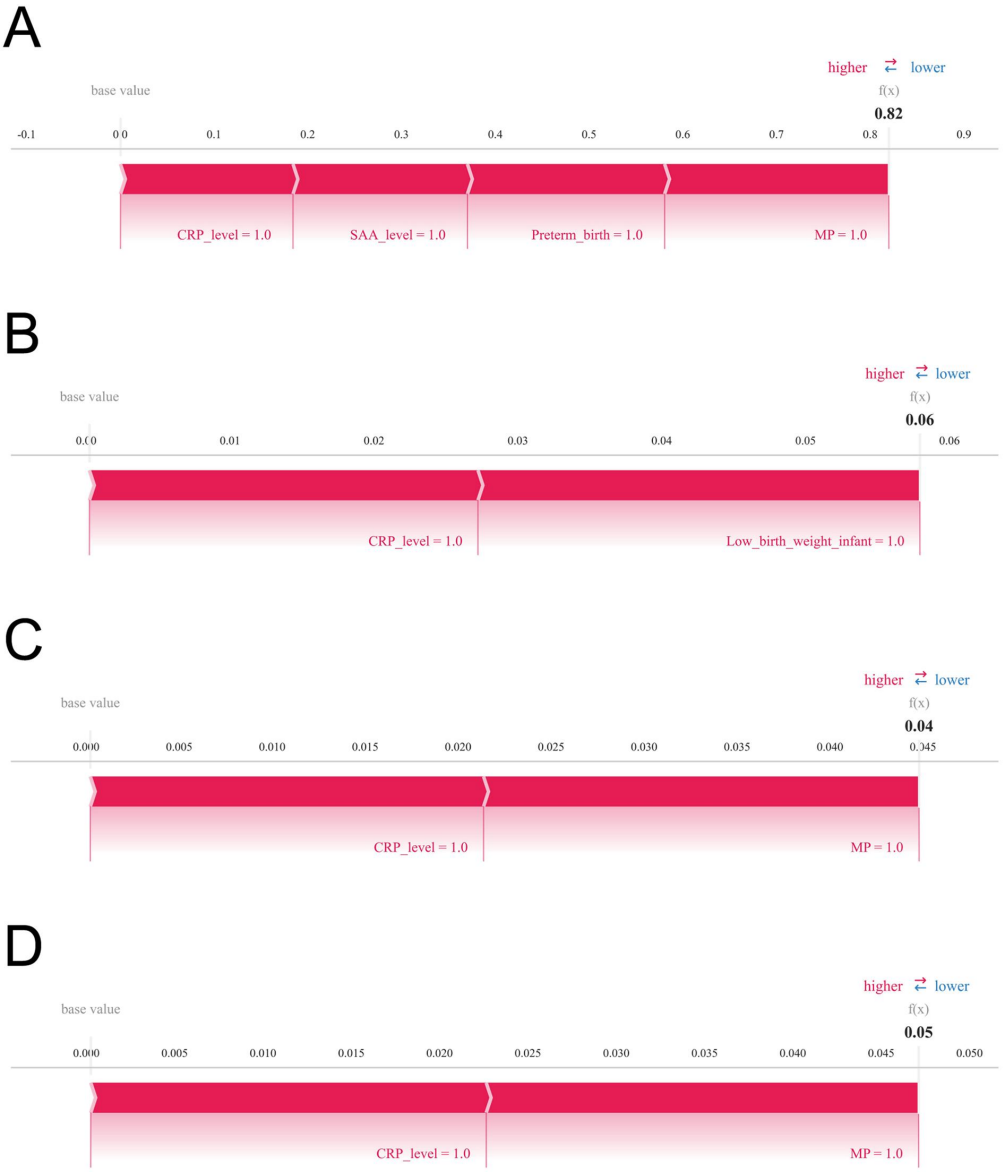
Illustrates SHAP force plots that provide individualized explanations of prediction outcomes. Variables are arranged horizontally based on their absolute impact magnitude, with blue bars indicating features that reduce predicted risk (negative SHAP values) and red bars indicating features that increase predicted risk (positive SHAP values). Panels **(A)** through **(D)** correspond to four representative patients, respectively.

To further assess the model's clinical interpretability and applicability, we examined individual prediction outcomes for four representative patients using SHAP force plots (Figures 7A–D). These plots illuminate patient-specific high-risk contributors and quantify their respective impact magnitudes. Patient 1: The model predicted a high probability (0.82) of developing infantile hemangioma, primarily driven by elevated CRP and SAA levels, preterm birth, and multiple pregnancy, indicating a high-risk profile. Patient 2: Predicted risk was low (0.06), with minor contributions from CRP levels and low birth weight, suggesting a limited cumulative effect of risk factors in this case. Patient 3: The model estimated a probability of 0.04, where CRP levels and multiple pregnancy were the main contributors, indicating a low overall risk. Patient 4: Predicted probability was 0.05, with CRP level and multiple pregnancy as the key contributing factors. Although classified as low risk, ongoing monitoring of these variables may be advisable. These individualized explanations demonstrate the capacity of the XGBoost model combined with SHAP analysis to enable precision risk stratification, thereby supporting nuanced and informed clinical decision-making.

## Discussion

In this study, we employed four widely used machine learning algorithms—RF, SVM, KNN, and XGBoost—to develop a clinical prediction model for infantile hemangioma. RF, which aggregates numerous decision trees via majority voting, exhibits robust noise tolerance and excels with high-dimensional data by effectively evaluating feature importance; however, it can struggle with capturing complex nonlinear interactions and is computationally intensive due to its intricate architecture (12, 21, 22). SVM constructs a maximal-margin hyperplane and performs well on high-dimensional, small-to-medium datasets but is sensitive to kernel selection and parameter tuning, with reduced efficiency on large datasets. KNN offers intuitive simplicity by predicting outcomes based on sample proximity, making it suitable for low-dimensional, small-sample contexts, but it suffers from the curse of dimensionality and high computational demands, limiting scalability. Conversely, XGBoost, an ensemble method leveraging gradient boosting, iteratively builds weak learners to capture complex nonlinear relationships efficiently. Its integrated regularization mitigates overfitting, while support for parallel computation and automatic handling of missing data enhances both accuracy and efficiency (23–25).

Our systematic model construction and evaluation revealed XGBoost's superior performance across multiple metrics. ROC analysis demonstrated outstanding predictive capability, with AUCs of 0.952 and 0.935 in training and validation cohorts, respectively, outperforming RF, SVM, and KNN. These values attest to its exceptional discriminative power in stratifying high- vs. low-risk patients. Calibration curves confirmed excellent concordance between predicted and observed probabilities, supporting the model's reliability in both risk stratification and probability estimation. Decision curve analysis further substantiated XGBoost's clinical utility, consistently yielding higher net benefits across a wide range of thresholds, underscoring its translational potential in clinical settings. K-fold cross-validation within the internal cohort reinforced these findings: XGBoost achieved a mean validation AUC of 0.9438 ± 0.0484, a test set AUC of 0.8366, and accuracy of 0.8943—surpassing RF (AUC = 0.8510 ± 0.1334, accuracy = 0.8415), SVM (AUC = 0.8326 ± 0.1362, accuracy = 0.9472), and KNN (AUC = 0.8466 ± 0.1243, accuracy = 0.8780). These results underscore XGBoost's superior discriminative capacity, accuracy, generalizability, and stability. External validation confirmed the model's robustness, with XGBoost achieving an AUC of 0.870, demonstrating adaptability to unseen data across different populations and clinical environments. Accordingly, XGBoost emerged as the optimal algorithm for predicting high-risk IH factors by effectively modeling nonlinearities, minimizing overfitting via regularization, and utilizing parallelism to optimize training efficiency—providing a solid foundation for early screening and individualized interventions.

Leveraging feature importance rankings from XGBoost, we explored key risk factors through SHAP analysis, focusing on two biological pathways implicated in IH pathogenesis: immune activation and hypoxic stress. SAA and CRP, acute-phase inflammatory markers, emerged as significant contributors, suggesting a pivotal role of immune responses in hemangioma development. Both SAA and CRP rise markedly during infection, tissue injury, or inflammation; notably, SAA may promote angiogenesis by facilitating endothelial cell migration and proliferation (26–28). Mechanistically, this likely involves activation of Toll-like receptors and NF-*κ*B signaling, upregulating pro-angiogenic mediators such as VEGF, thereby driving hemangioma formation (8, 29–31). Inflammation can also alter the immune microenvironment and impair T cell–mediated surveillance, allowing aberrant endothelial proliferation to evade immune detection and promote tumor growth. Given the immaturity of the neonatal immune system, perinatal inflammatory stimuli—such as maternal immune activation or infection—may predispose infants to immune dysregulation and abnormal angiogenesis.

SHAP-based analyses of individual risk profiles further underscored hypoxic stress as a key pathogenic mechanism. For example, Patient 1's risk was influenced by multiple pregnancy, prematurity, and low birth weight—all associated with intrauterine or perinatal hypoxia. Hypoxia activates hypoxia-inducible factor-1α (HIF-1α), which enhances VEGF and other angiogenic factors, promoting endothelial proliferation, migration, and aberrant vascular formation (32, 33). Preterm and low birth weight infants often experience systemic hypoxia due to placental insufficiency or immature pulmonary function, stimulating angiogenesis and aberrant endothelial progenitor cell mobilization, accelerating hemangioma growth. Hypoxia may also impair immune maturation, amplifying inflammation and immune dysregulation, synergistically fostering tumor progression (34–38).

The predictive model developed herein offers a complementary tool for the early identification of high-risk IH in neonates and can be seamlessly integrated into clinical screening workflows. During birth or early follow-up, infants’ perinatal data and serum immune–inflammatory biomarkers can be collected, and the model employed to stratify them into high- and low-risk groups. High-risk infants may be prioritized for imaging evaluations (e.g., ultrasound or MRI) to confirm diagnosis and facilitate timely intervention, whereas low-risk infants can continue with standard follow-up, thereby optimizing allocation of healthcare resources. Beyond guiding clinicians in devising personalized monitoring protocols and health education strategies—enhancing early detection and minimizing delayed diagnoses—the model mitigates unnecessary testing and associated economic burdens while maintaining safety. At a public health level, it provides quantitative evidence to inform newborn screening policies and health management strategies. Conceptually, by integrating immune–inflammatory biomarkers with machine learning, the model affords novel insights into IH pathogenesis and informs future strategies for early prediction and personalized intervention. Consequently, the principal beneficiaries encompass neonates and their families, clinicians, and public health authorities, while the research community gains a broadly applicable framework for predictive modeling and decision support.

Previous studies typically involved small sample sizes, lacked systematic integration of perinatal and immune–inflammatory indicators, and largely relied on conventional statistical methods (39). This study uniquely integrates immune-related biomarkers into the IH risk prediction framework, overcoming limitations of prior research that focused mainly on clinical or imaging features. Incorporating immunological parameters enhances biological interpretability and elucidates disease mechanisms. The comprehensive comparison and validation of multiple machine learning models across internal and external cohorts demonstrate the XGBoost model's superior stability, reproducibility, and clinical applicability. Multi-dimensional evaluation—including calibration and decision curve analyses—further reinforces model reliability and translational potential. Nonetheless, limitations exist. First, data were sourced from a single center; despite external validation, limited geographic and demographic diversity may restrict generalizability. Second, immune biomarkers such as SAA and CRP are susceptible to confounding factors like infection or medication, potentially introducing variability; future studies should consider dynamic monitoring to improve precision. Third, despite SHAP's interpretability advantages, the inherent “black-box” nature of complex models like XGBoost may impede clinical transparency and acceptance. Future research should integrate more interpretable approaches and involve larger, multicenter datasets to validate robustness and facilitate clinical integration. With regard to class imbalance, the proportion of IH cases in this study was approximately 5.5%, reflecting a moderate degree of imbalance. We did not implement techniques such as SMOTE, undersampling, or class weighting, guided by the following considerations: first, ensemble algorithms like XGBoost and Random Forest inherently possess strong robustness to class imbalance, mitigating its effects through internal sample weighting and structural mechanisms; second, stratified random sampling was applied to preserve consistent class distributions between training and testing sets, coupled with ten-fold cross-validation to enhance model stability and generalizability. Nonetheless, the absence of dedicated imbalance-handling strategies may have constrained the performance of certain models—particularly SVM and KNN—in accurately identifying minority-class samples, representing a limitation of this study. Future investigations will consider incorporating SMOTE, class weighting, and related approaches, systematically evaluating their influence on model performance. Furthermore, due to limitations of the medical record system, detailed data on pregnancy-related pathological factors could not be comprehensively obtained or presented, representing an additional study limitation. Moreover, different IH subtypes may have distinct pathogenic mechanisms and risk factors, which could lead to variability in the predictive performance of the model. However, in this study, the total IH sample comprised only 81 cases, with limited numbers in each subtype (superficial, *n* = 50; deep, *n* = 20; mixed, *n* = 11). Conducting subgroup analyses under these conditions may result in insufficient statistical power, precluding reliable conclusions. This represents a major limitation of the current study. In future research, we plan to perform subtype-specific analyses in larger, multicenter cohorts to validate the model's predictive performance across different IH subtypes.

## Conclusion

This study systematically evaluated the predictive performance of four machine learning algorithms for high-risk infantile hemangioma, demonstrating that XGBoost significantly outperformed the others in accuracy, robustness, and generalizability. Utilizing SHAP analysis, we elucidated the relative importance of key risk factors, identifying serum amyloid A SAA levels, low birth weight, VEGF expression, multiple gestations, prematurity, and CRP levels as the most prognostically influential variables. These findings provide critical insights for early clinical identification of high-risk infants and lay the foundation for developing personalized intervention strategies.

## Data Availability

The original contributions presented in the study are included in the article/Supplementary Material, further inquiries can be directed to the corresponding author.

## Supplementary material

Supplementary Table S1 Comparison of clinical indicators between patients with hemangioma and those without hemangioma.

Supplementary Table S2 Raw Data.
